# Fe- and S-Modified BiOI as Catalysts to Oxygen Evolution and Hydrogen Evolution Reactions in Overall Photoelectrochemical Water Splitting

**DOI:** 10.3390/ma17010006

**Published:** 2023-12-19

**Authors:** Yu Lei, Hongdian Chen, Chenyang Shu, Changguo Chen

**Affiliations:** 1 College of Chemistry and Chemical Engineering, Chongqing University, Chongqing 401331, China; 2 Chongqing Key Laboratory of Materials Surface & Interface Science, Chongqing University of Arts and Sciences, Chongqing 402160, China; 18323079864@163.com (H.C.); 13606109577@163.com (C.S.)

**Keywords:** bismuth oxyiodide, iron modification, sulfurization, overall water splitting, photoelectrochemical catalysis

## Abstract

Developing catalysts with superior activity to hydrogen evolution reaction (HER) and oxygen evolution reaction (OER) is equally important to the overall photoelectrochemical water splitting to produce hydrogen. In this work, bismuth oxyiodide (BiOI), iron-modified bismuth iodide Fe/BiOI, and the sulfurized S-Fe/BiOI were prepared using the solvothermal method. The three materials all have good absorption ability for visible light. The photoelectrochemical catalytic activity of BiOI to oxygen evolution reaction (OER) is significantly enhanced after iron modification, while the sulfurized product S-Fe/BiOI exhibits better catalytic activity to hydrogen evolution reaction (HER). Hence, OER and HER can be simultaneously catalyzed by using Fe/BiOI and S-Fe/BiOI as anodic and cathodic catalysts to facilitate the overall photoelectrochemical water splitting process.

## 1. Introduction

It is urgent to develop renewable, environmentally friendly, and cheap energy resources and technologies due to the depleting fossil fuels and the environmental issues associated with the combustion of fossil fuels. When 1 mole of hydrogen reacts with oxygen, 285.83 kJ mol^−1^ of heat can be released, which means hydrogen is considered to be a promising fuel in the 21st century [1,2]. However, the large scale of application of hydrogen energy is still not realized even after a quarter of the 21st century has passed. One of obstacles is that the sustainable production of hydrogen is not economically achieved. Nowadays, hydrogen is mainly the byproduct of reformed organic compounds, in which fossil fuels (such as methane) are consumed and large amounts of carbon dioxide are released. Hence, the method is also not sustainable [3], while splitting water by electrolysis, photocatalysis, and photoelectrolysis is setfor great expectations [4,5,6].

Oxygen evolution reaction (OER) and hydrogen evolution reaction (HER) separately occur on the anode and cathode of an electrolyte cell during the catalytic electrolysis of water. HER cannot effectively process if OER was hindered. Hence, OER and HER are equally important to the hydrogen production by electrolysis of water. Unfortunately, precious metal-based catalysts are necessary for both reactions. For example, Pt-based materials are better catalysts to the HER, and RuO_2_ and IrO_2_ are the benchmark catalysts to the OER [7,8]. The scarcity and high price of these precious metals restrict the wide application of these catalysts. Developing nonprecious metal-based catalysts with a high performance and low cost is inevitable to the practical application of electrolysis of water. Recently, the oxides/hydroxides of Ni, Fe, and Co have been found to have superior OER activity [9,10,11,12], while some oxides and sulfides of these transition metal elements show appreciable HER activity [13,14,15]. These investigations provide promising options for the electrolysis of water. Meanwhile, producing hydrogen using the photocatalysis and photoelectrolysis methods is attracting intensive attentions since Fujishima and Honda published their research in 1972 [16].

Many photocatalysts have been successfully developed, which makes it possible to produce hydrogen under mild conditions with green and sustainable strategies. Among these, transition metal sulfides and composites with heterostructures exhibit special photocatalytic performance due to the easy generation of photo charges and the inhibited recombination of electrons with holes [17,18]. For example, an N-doped C-CoS_2_@CoS_2_/MoS_2_ nanopolyhedron with hierarchical yolk-shelled structures was prepared as a bifunctional catalyst for photovoltaics and hydrogen evolution using an ion-exchange strategy with a low onset potential and a small Tafel slope [19]. A FeOOH/Au/BiVO_4_ photoanode was fabricated through dual modification with Au nanoparticles and FeOOH nanoneedles on nanoporous BiVO_4_ surface. Benefiting from the enhanced visible light absorption of Au nanoparticles and the extracted photogenerated holes of FeOOH, the catalyst displayed a photocurrent density of 4.64 mA cm^2^ at 1.23 VRHE, which was 3.74 times higher than the pristine BiVO_4_ [20]. A kermesinus bismuth oxyiodide (BiOI) was prepared through a UV-Vis-Light driving method to introduce oxygen vacancies (OVs) onto the surface of BiOI, exhibiting a remarkably improved hydrogen production rate [21]. Among the catalysts, the bandgap of p-type semiconductor BiOI is 1.7~1.9 eV, and visible light with a wavelength lower than 653 nm can be well absorbed by BiOI, exhibiting the potential prospects in the field of photoenergy transformation [22,23,24,25]. The catalytic activity of BiOI can be enhanced by constructing heterostructures with other material. For example, the photocatalytic activity and stability of BiOI in organic degradation was significantly enhanced by covering it with Bi_2_O_2_CO_3_ [26]. The adsorption capacity of visible light and the charge separation efficiency of BiOI were improved by compositing BiOI with kaolinite [27] and TiO_2_ [28]. Moreover, the performance of other photocatalysts such as g-C_3_N_4_ can also be boosted by the modification of BiOI, by which the conversion efficiency of CO_2_ reduction to CH_4_ was increased to 39.43 µmol g^−1^ from 4.09 µmol g^−1^ of the bulk g-C_3_N_4_[29]. Meanwhile, bismuth sulfide (Bi_2_S_3_) is an n-type semiconductor with a narrow bandgap (1.3~1.7 eV), and it is also gaining special attention in hydrogen production due to its favorable photosensitivity, inexpensiveness, nontoxicity, and so on [30,31,32,33,34].

In this work, iron-modified Fe/BiOI was prepared using a solvothermal method and then was further sulfurized by a second solvothermal treatment to fabricate S-Fe/BiOI in which partial Bi was transferred to Bi_2_S_3_ to achieve the couple of electrochemical catalyst with photocatalyst. The Fe/BiOI exhibits enhanced photoelectrochemical activity of the OER, while S-Fe/BiOI shows good activity in the HER. An overall water splitting system can be constructed by using Fe/BiOI and S-Fe/BiOI as the catalyst on the anode (OER) and cathode (HER), respectively. The electrolysis voltage is apparently decreased under visible light irradiation, and oxygen and hydrogen are easily bubbled on the corresponding electrodes.

## 2. Experimental Procedure

### 2.1. Preparation of Catalysts

Reagents were purchased from Aladdin (China) without further purification.

**Figure 1:**
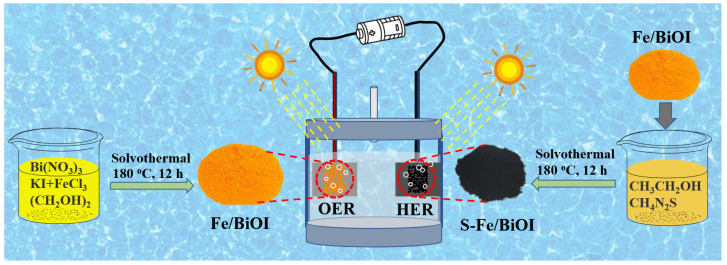
Schematic illustration for the preparation of the modified BiOI catalysts.

The preparation process of catalysts is schematically diagramed in Figure 1. Firstly, 0.010 mol bismuth nitrate (Bi(NO_3_)_3_·5H_2_O, 4.9 g) was dispersed in 70 mL ethylene glycol (C_2_H_6_O_2_) under stirring, then 0.010 mol KI (1.6 g) was added to the solution. The solution was equally divided into two parts of 35 mL. Then, 0.2 mmol FeCl_3_·6H_2_O (0.056 g, at a mole ratio 1:50 between Fe:Bi) was added into one part. The two solutions were transferred into Teflon-lined stainless-steel autoclaves, and solvothermally reacted at 180 °C for 12 h. After the reaction, the autoclaves were cooled naturally. The obtained precipitates were washed with de-ionized water and ethanol, and collected by centrifugation, followed by drying in air at 60 °C. The two products were named BiOI and Fe/BiOI. To achieve the transformation from BiOI to Bi_2_S_3_, sulfurized S-Fe/BiOI was further fabricated by ultrasonically dispersing 0.5 g Fe/BiOI and 0.2 g thiourea (CH_4_N_2_S) in 50 mL ethanol, followed by a secondary solvothermal treatment at 180 °C for another 12 h, washing and drying.

To explore the effects of the contents of Fe and S, 0.05 mmol, 0.1 mmol, 0.2 mmol, 0.4 mmol, and 0.6 mmol FeCl_3_·6H_2_O were used to fabricate the series of Fe/BiOI catalysts (named 0.05Fe/BiOI, 0.1Fe/BiOI, etc.) Among these catalysts, 0.2Fe/BiOI showed the better OER and HER activities and was further sulfurized with 0.03 g, 0.06 g, 0.1 g, and 0.2 g thiourea (named 0.03S-Fe/BiOI, 0.06S-Fe/BiOI, etc.)

### 2.2. Physical Characterizations

X-ray diffraction (XRD, DX-2700BH, Dandong Haoyuan, Dandong, China) was used to analyze the phase structure of catalysts. X-ray photoelectron spectroscopy (XPS) was recorded on the Kratos XSAM800 (Britain) spectrometer. The morphologies of the catalysts were observed using a scanning electron microscope (SEM, Gemini SEM 300, Zeiss, Jena, Germany) with attached energy dispersive spectroscope (EDS, X-MAX, Oxford, UK), and high-resolution transmission electron microscopy (HRTEM) on Tecnai TEM G2F30x microscope (FEI, Hillsboro, OR, USA). The light response was measured by UV-Vis diffuse reflectance spectra (UV-Vis DRS, UH4150, HITACHI, Chiyoda City, Japan).

### 2.3. Photoelectrochemical Catalysis Measurements

Photoelectrochemical (PEC) measurements were carried out on a three-electrode system in 0.5 M Na_2_SO_4_ solution (pH ≈ 7) in a quartz cell. A platinum foil and a saturated calomel electrode (SCE) were used as the counter electrode and reference electrode. The measured potential was calibrated with respect to the reversible hydrogen electrode (RHE, *E*_RHE_ = *E*_SCE_ + 0.0592 pH + 0.241 V ≈ *E*_SCE_ + 0.6554 V). The working electrode was prepared as follows: 30 mg catalyst was ultrasonically dispersed in 0.5 mL 0.5% Nafion solution (3:7 of volume ratio between water and ethanol) for 12 h, then 4 × 10 μL slurry was dropped on the two sides of a piece of carbon paper (1 cm × 1 cm, TGP-H-060, TORAY, Tokyo, Japan), and dried naturally in air. PEC performances were measured under simulated sunlight irradiation from a 500 W Xe lamp with a 400 nm filter at 50 mW cm^−2^ (XM-500, NBeT, Beijing, China). The catalytic activities in the HER and OER were evaluated with linear sweep voltammetry (LSV) at 10 mV s^−1^. The overall photoelectrochemical water splitting was measured using the chronopotentiometry (*i*-*t* curve) method at 1.6 V (vs. RHE).

## 3. Results and Discussion

**Figure 2:**
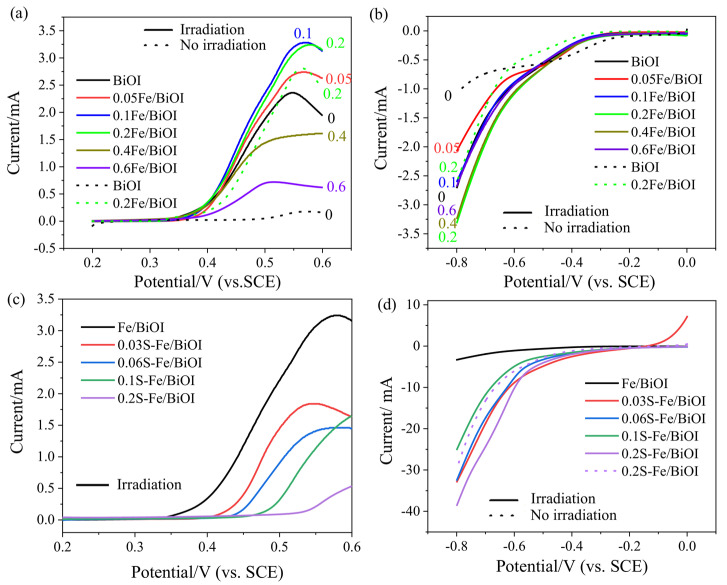
Effects of Fe content on (**a**) OER and (**b**) HER; effects of S content on (**c**) OER and (**d**) HER.

The effects of the contents of Fe and S on photoelectrochemical catalytic activities to oxygen evolution reaction (OER) and hydrogen evolution reaction (HER) in water splitting were first investigated using the linear sweep voltammetry (LSV) method. As presented in Figure 2a, the OER current on the blank BiOI was obviously increased under irradiation of visible light, revealing the superior photochemical activity of BiOI. With the addition of 0.05 mmol, 0.1 mmol, or 0.2 mmol Fe, the photochemical OER current of the Fe/BiOI was further increased, and then was decreased with excessive addition of Fe (0.4 mmol or 0.6 mmol). The data of 0.2Fe/BiOI also shows that the OER current is sharply higher than that of BiOI without irradiation, indicating that the Fe species in the Fe/BiOI mainly exhibits as an electrochemical catalyst to the OER. The effect of Fe on the HER is presented in Figure 2b. As observed, the photocurrent was increased with a slight level, showing that the Fe species in the Fe/BiOI is inert in the HER. Comprising the performances on OER and HER, 0.2Fe/BiOI was further sulfurized by thiourea to achieve the transformation of BiOI to Bi_2_S_3_ to construct the coupling of the electrochemical catalyst with the photocatalyst. As displayed in Figure 2c,d, the OER activity of S-Fe/BiOI is deteriorated after sulfurization processing, but the photocurrent of HER is boosted from 3.3 mA@-0.8 V of Fe/BiOI to 39 mA@-0.8 V of 0.2S-Fe/BiOI due to the generation of Bi_2_S_3_. Hence, it is an adoptable strategy to construct a device for the overall photoelectrochemical water splitting by using Fe/BiOI as the OER catalyst on the anode and S-Fe/BiOI as the HER catalyst on the cathode.

**Figure 3:**
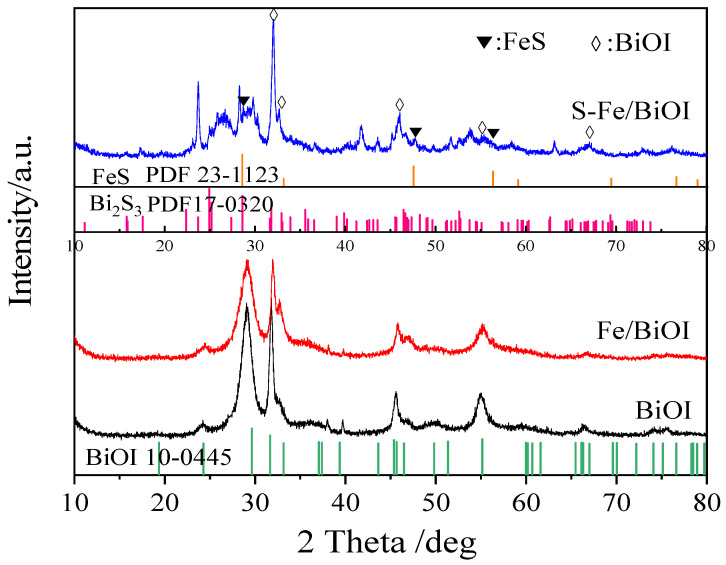
X-ray diffraction patterns of BiOI, Fe/BiOI, and S-Fe/BiOI.

To understand the mechanism of the enhanced OER activity of Fe/BiOI and the enhanced HER activity of S-Fe/BiOI, the optimized 0.2Fe/BiOI (named Fe/BiOI below) and 0.2S-Fe/BiOI (named S-Fe/BiOI below) were thoroughly characterized and analyzed. The XRD patterns of the catalysts are presented in Figure 3. As observed, the strong diffraction peaks of the pristine BiOI at 29.6°, 31.7°, 45.4°, and 55.2° can be attributed to crystal plane (102), (110), (200), and (212), which are in accordance with the tetragonal BiOI (PDF 10-0445). No Fe-related peaks can be found in the XRD pattern of Fe/BiOI with the doping of Fe. The possible reason is that the content of Fe is low (Bi:Fe = 50:1 in mole) and the diffraction intensity of Fe-related substances is too weak to be checked [35,36]. However, the broadened diffraction peaks of BiOI in Fe/BiOI suggests the possible doping of Fe-related species. After sulfurizing by thiourea, the signals of FeS (PDF 23-1123) and Bi_2_S_3_ (PDF 17-0320) can be observed in the XRD pattern of S-Fe/BiOI, revealing that the Fe oxides were co-deposited with BiOI, and the thiourea can react with the Fe- and Bi-related species in the secondary solvothermal treatment. The generation of Bi_2_S_3_ would endow the composite S-Fe/BiOI with the enhanced catalytic activity of the HER.

**Figure 4:**
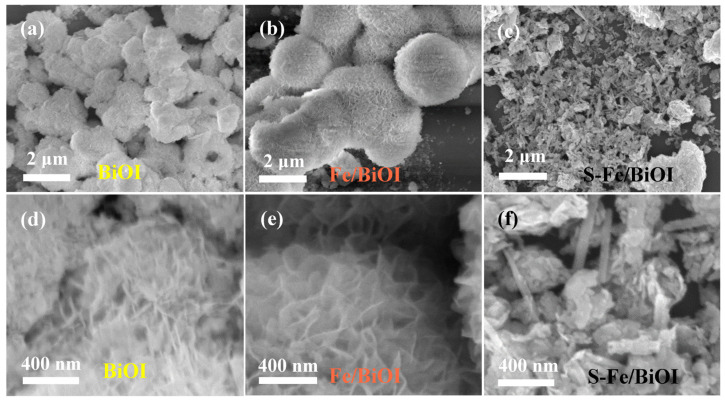
SEM images of (**a**,**d**) BiOI, (**b**,**e**) Fe/BiOI, (**c**,**f**) S-Fe/BiOI at different magnification.

The morphologies of BiOI, Fe/BiOI, and S-Fe/BiOI were observed by SEM. As shown in Figure 4, the pristine BiOI and Fe/BiOI exhibit a flower-like hierarchical architecture aggregated from two-dimensional nanosheets, showing that the trace doping of Fe does not change the microstructure of BiOI. However, the color of BiOI was changed from yellow to orange as shown in Figure 1, further demonstrating that Fe atoms enter the structure of BiOI. The two-dimensional nanosheets of BiOI can facilitate the diffusion of the solution and the exposure of active sites, both being beneficial to enhance the catalysis process. After being sulfurized, the flower-like spheres of Fe/BiOI were apparently transferred to nanorods and particles in S-Fe/BiOI, which is the typical morphology of Bi_2_S_3_ with an orthorhombic crystal system, verifying that thiourea reacted with BiOI to generate Bi_2_S_3_. The signal of BiOI was also found in the XRD pattern of S-Fe/BiOI, showing that BiOI coexists in the rods of Bi_2_S_3_ and provides possible bifunctional catalytic activities to the OER and HER of the S-Fe/BiOI.

**Figure 5:**
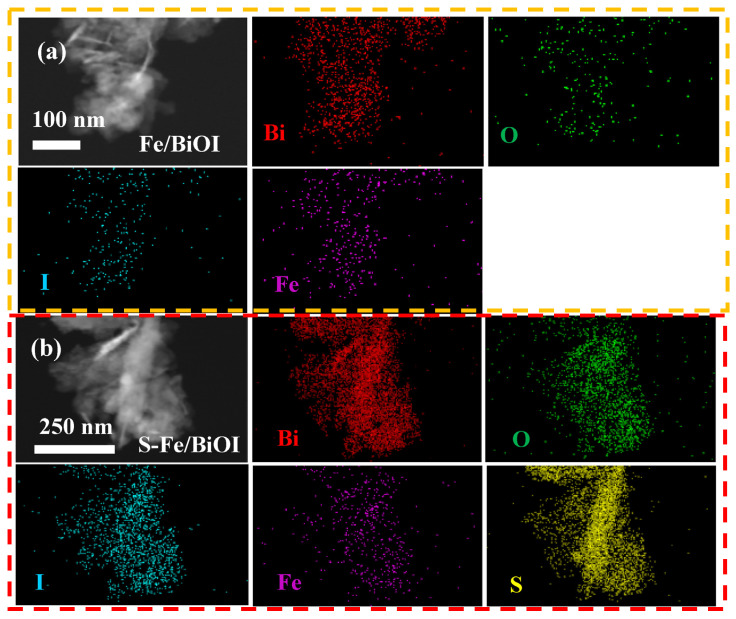
EDS mapping, (**a**) Fe/BiOI, (**b**) S-Fe/BiOI.

**Figure 6:**
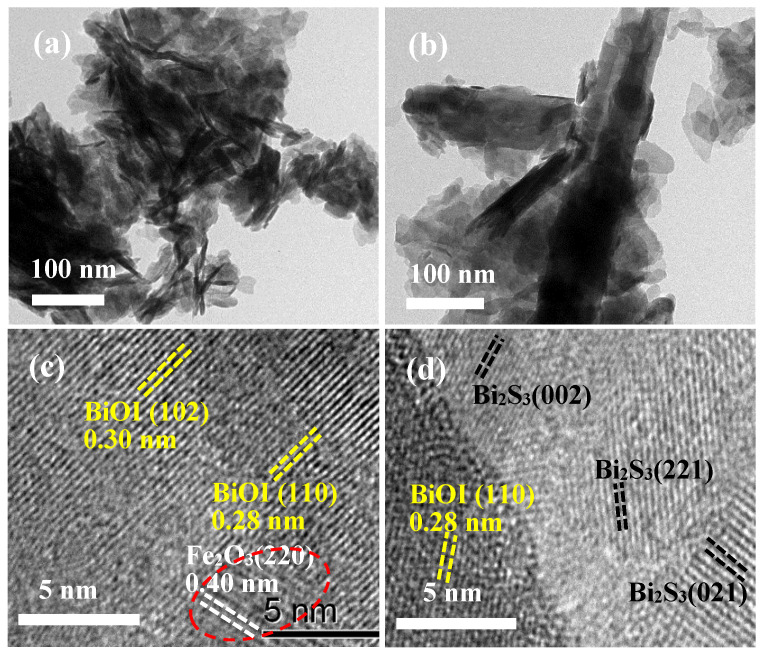
TEM images of (**a**) Fe/BiOI, (**b**) S-Fe/BiOI; HRTEM images of (**c**) Fe/BiOI, (**d**) S-Fe/BiOI.

The elemental compositions of catalysts were mapped by energy dispersive spectroscopy (EDS). As shown in Figure 5a, Fe was found to be uniformly dispersed in Fe/BiOI, indicating the co-deposition of Fe-related species with BiOI even though their diffraction peaks were not directly distinguished in the XRD pattern of Fe/BiOI. As displayed in Figure 5b, the S element is intensively distributed in the EDS mapping of S-Fe/BiOI beside Fe, revealing that the BiOI reacted well with thiourea to achieve the generation of bismuth sulfide. The nanosheets in Fe/BiOI and the rods in S-Fe/BiOI were further demonstrated in TEM images as presented in Figure 6a,b, also showing the flower-like morphology for BiOI and the rod-like morphology for S-Fe/BiOI. The physical phase of the catalyst was further checked by HRTEM. The 0.30 nm and 0.28 nm of lattice distances in Fe/BiOI (Figure 6c) refer to the d-spacing of (102) and (110) facets of BiOI (PDF 10-0445), while the lattice fringes with 0.40 nm of distance correspond to the (220) plane of Fe_2_O_3_ (PDF 16-0653) and verify the generation of Fe oxide which plays the role of catalyzing the oxygen evolution reaction. The HRTEM of S-Fe/BiOI (Figure 6d) also demonstrates the lattice fringes of BiOI and Bi_2_S_3_ (PDF 17-0320) which provides the catalytic active sites for hydrogen evolution reaction.

**Figure 7:**
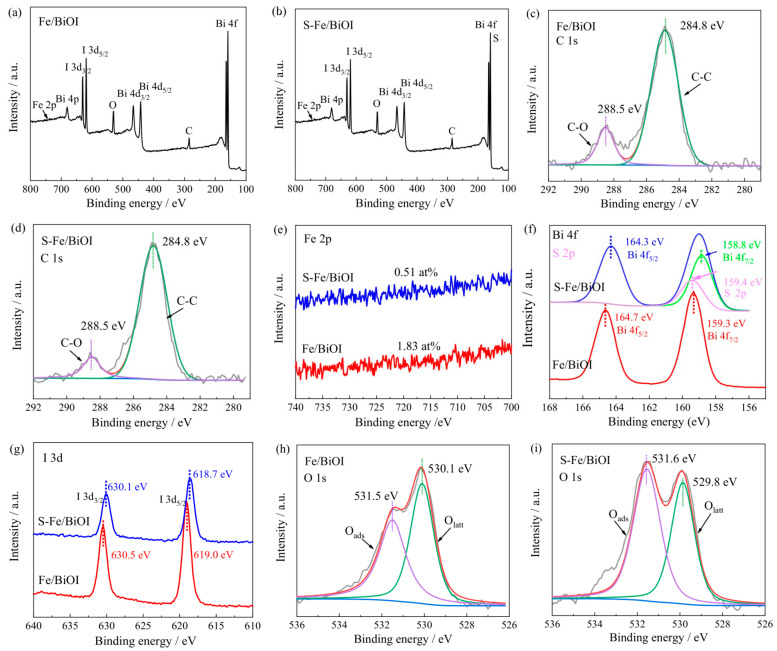
XPS survey spectrum, (**a**) Fe/BiOI, (**b**) S-Fe/BiOI; high-resolution XPS spectrum, (**c**,**d**) C 1s, (**e**) Fe 2p, (**f**) Bi 4f, (**g**) I 3d, (**h**,**i**) O1s.

The composition and the electronic state of the elements of the samples were characterized by X-ray photoelectron spectroscopy (XPS). As presented in Figure 7a, Bi, O, I, and C can be found in the survey spectrum of Fe/BiOI and additional S in the spectrum of S-Fe/BiOI (Figure 7b) [37]. The C atoms should be from the adsorbed solvent for which the C-C and C-O structures can be fitted in the high-resolution XPS spectra of C 1s (Figure 7c,d) [38]. The XPS intensities of Fe in the spectra of Fe/BiOI and S-Fe/BiOI are weak due to its low content with 1.83 at% and 0.51 at% of the atomic ratio in the catalysts (Figure 7e). The high-resolution XPS spectra of Bi 4f, I 3d, and O 1s were also analyzed. As presented in Figure 7f, the two peaks at 159.3 eV and 164.7 eV refer to Bi 4f_7/2_ and Bi 4f_5/2_, indicating that Bi exists as Bi^3+^ in Fe/BiOI [39]. The binding energies of the two peaks negatively shift to 158.8 eV and 164.3 eV in S-Fe/BiOI due to the Bi-O being replaced by Bi-S from Bi_2_S_3_ [40,41]. The shift in binding energy can also be observed in the XPS spectrum of I 3d (Figure 7g) and O 1s (Figure 7h,i). [42,43] The binding energies of I 3d_5/2_ and I 3d_3/2_ are negatively moved to 618.7 eV and 630.1 eV from 619.0 eV and 630.5 eV while the binding energy of the lattice oxygen from Bi-O in the XPS spectrum of O 1s is shifted to 529.8 eV from 530.1 eV after sulfurizing the Fe/BiOI to S-Fe/BiOI, respectively. As a result, more electrons can be accumulated by Bi_2_S_3_ in S-Fe/BiOI to boost the catalytic activity to the HER.

**Figure 8:**
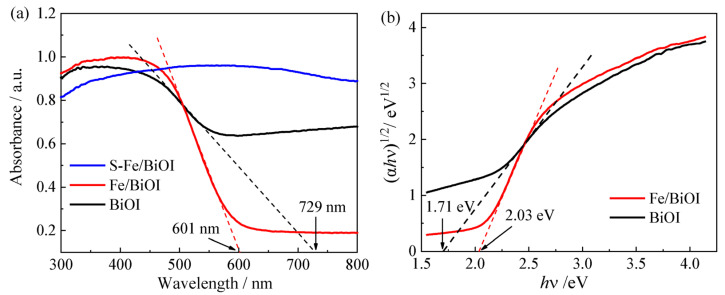
(**a**) UV-Vis diffuse reflectance spectra, (**b**) Kubelka–Munk plots.

The light absorption performance of BiOI, Fe/BiOI, and S-Fe/BiOI were detected by UV-Vis diffuse reflectance spectra (DRS). As shown in Figure 8a, both the pristine BiOI and Fe-doped Fe/BiOI show good absorption capacity in the visible region with an absorption edge at ~600 nm, but the doping of Fe decreases the absorption capacity of Fe/BiOI at the long-wave red light region. Furthermore, the absorption ability of the sulfurized S-Fe/BiOI was enhanced without an obvious absorption edge with the color of Fe/BiOI changed to black. The band gaps of BiOI and Fe/BiOI were estimated on the Kubelka–Munk transformed reflectance spectra (Figure 8b), being 1.71 eV for BiOI and 2.03 eV for Fe/BiOI. The slightly increased bandgap of Fe/BiOI also demonstrates the generation of Fe_2_O_3_ which has a greater bandgap (2.0~2.2 eV) than that of BiOI (1.7~1.9 eV). The increased bandgap can prevent the recombination of photogenerated charges, allowing the electrons or holes to be effectively utilized [18].

**Figure 9:**
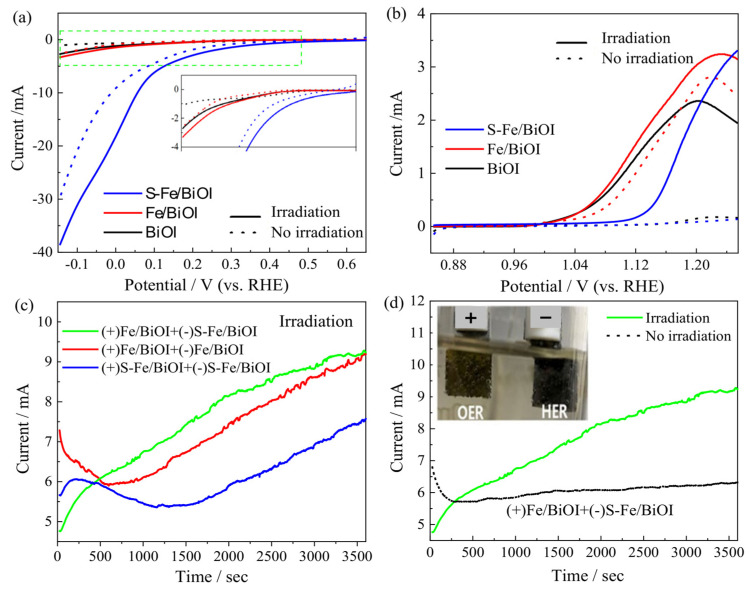
Photoelectrochemical catalytic performance, (**a**) LSV curves of HER, (**b**) LSV curves of OER, (**c**) *i*-*t* curves of overall water splitting with different combinations of catalysts, (**d**) light response of (+)Fe/BiOI+(−)S-Fe/BiOI system.

The photoelectrochemical catalytic activities of the BiOI, Fe/BiOI, and S-Fe/BiOI in the HER and OER were systematically compared. As presented in Figure 9a, all HER photocurrents on BiOI, Fe/BiOI, and S-Fe/BiOI increased under visible light irradiation, showing that the HER is promoted by photogenerated electrons. However, the current on S-Fe/BiOI is sharply increased and the HER overpotential is reduced even without irradiation, revealing that both BiOI and iron oxides are not good HER catalysts, but Bi_2_S_3_ is a superior electrocatalyst for the HER. Furthermore, the HER current is increased on S-Fe/BiOI under irradiation, indicating Bi_2_S_3_ is also active in utilizing the energy of visible light to boost the HER process. Hence, Bi_2_S_3_ is an excellent photoelectrochemical catalyst for the HER.

Figure 9b displays the LSV curves of the OER. As observed, the OER current is low on BiOI without irradiation, showing worse electrochemical catalytic activity of BiOI for the OER. But the OER current on BiOI is obviously increased under irradiation, indicating the superior photochemical activity of BiOI in the OER. With the doping of Fe, the resultant Fe/BiOI exhibits enhanced OER electrochemical activity with high OER current and low overpotential, demonstrating the superior activity of iron oxide as displayed in the electrochemical OER process. The OER performance of Fe/BiOI is further boosted under irradiation, suggesting a synergistic effect between photoactive BiOI and electrochemical-active iron oxide to endow Fe/BiOI with the better photoelectrochemical catalytic activity to the OER. The OER activity of S-Fe/BiOI is deteriorated due to the sulfurization of iron oxide. Hence, both the Fe/BiOI and S-Fe/BiOI are not bifunctional catalysts for OER and HER, but the overall photoelectrochemical water splitting can be achieved by separately using Fe/BiOI on the anode as an OER catalyst and S-Fe/BiOI on the cathode as an HER catalyst.

The overall photoelectrochemical water splitting was carried out using the chronopotentiometry (*i*-*t* curve) method at 1.6 V on a two-electrode system with different combinations of BiOI, Fe/BiOI, and S-Fe/BiOI. As presented in Figure 9c, the photoelectrochemical current of water splitting on the system when using S-Fe/BiOI as the anode catalyst and cathode catalyst ((+)S-Fe/BiOI+(−)S-Fe/BiOI) is lower due to poor the OER activity of the S-Fe/BiOI catalyst. Whereas the photoelectrochemical current on the system when using Fe/BiOI as the anode catalyst and S-Fe/BiOI as the cathode catalyst ((+)Fe/BiOI+(−)S-Fe/BiOI) is obviously increased, showing the best performance for the overall photoelectrochemical splitting of water. As shown in Figure 9d, after establishing the stability of mass diffusion in the catalysts in the initial stage, the current on the ((+)Fe/BiOI+(−)S-Fe/BiOI) system is higher under irradiation than that without irradiation, revealing that the superior catalytic activities of Fe/BiOI in the OER and S-Fe/BiOI in the HER can be well realized in a practical hydrogen production.

## 4. Conclusions

In summary, photochemical catalysts to the OER and HER were successfully prepared by modifying BiOI with superior photocatalytic activity. Firstly, BiOI was prepared using a solvothermal method. With the doping of a trace of Fe, the resultant Fe/BiOI exhibited enhanced photoelectrochemical catalytic activity to the OER and can be utilized on the anode of photochemical water splitting. By further reacting Fe/BiOI with thiourea, the OER activity of the obtained S-Fe/BiOI is deteriorated, but the HER activity is sharply boosted with the transformation from BiOI to Bi_2_S_3_ which can act as an HER catalyst. An overall photoelectrochemical water splitting system can be effectively constructed by separately using Fe/BiOI on the anode as an OER catalyst and S-Fe/BiOI on the cathode as an HER catalyst. A higher photoelectrochemical current can be released under irradiation of visible light, showing the accelerating rate of water splitting and the promising prospect of the field of hydrogen production.

## Data Availability

Data are contained within the article.
